# Well-being trajectories and dynamic resource shifts in the transitions of retirement: a longitudinal study of Taiwanese older adults

**DOI:** 10.3389/fpsyg.2025.1449442

**Published:** 2025-06-27

**Authors:** Wan-Chen Hsu, Nuan-Ching Huang, Susan C. Hu

**Affiliations:** ^1^Department of Public Health, College of Medicine, National Cheng Kung University, Tainan, Taiwan; ^2^Department of Public Health, College of Health Care and Management, Chung Shan Medical University, Taichung, Taiwan; ^3^Department of Family and Community Medicine, Chung Shan Medical University Hospital, Taichung, Taiwan; ^4^Department of Public Health, College of Medicine, National Cheng Kung University, Tainan, Taiwan

**Keywords:** well-being, trajectory, retirement transition, adjustment, pension, latent growth mixture modeling (LGMM), dynamic resource-based model

## Abstract

**Background:**

Previous literature highlights the heterogeneity of retirement adjustment, emphasizing various life experiences among retirees. The Dynamic Resource-Based Model provides an integrated framework to examine these differences, linking retirement adjustment to subjective well-being. This study applied the framework to Taiwan’s unique sociocultural context, in which distinct cultural values and retirement policies may create different adaptation patterns from those observed in Western societies. Thus, the main purposes of the study are to examine the heterogeneity in subjective well-being trajectories among Taiwanese retirees and focus on how various resources influence these diverse adaptation patterns within this specific cultural environment.

**Method:**

This study utilized six waves of datasets from the Taiwanese Longitudinal Study on Aging (TLSA) spanning from 1996 to 2015, with 1,329 valid participants aged 50 and above in the analysis. Retirement was defined as the initiation of pension receipt during the observation period. Subjective well-being was measured using a 10-item Life Satisfaction Index. We first employed a Latent Growth Mixture Model (LGMM) to identify distinct trajectories of subjective well-being. Then, we applied the generalized linear model (GLM) to examine how physical, financial, cognitive, emotional, and social resources influence these trajectories across the following three stages: pre-retirement, transition, and post-retirement.

**Results:**

Our findings revealed two main trajectories of subjective well-being during retirement: a “High-Increase” group and a “Low-Decline” group. Compared with the “Low-Decline” group, individuals in the “High-Increase” group exhibited better health, stronger economic status, greater social participation, more family support, and higher educational attainment. In addition, older adults with fewer illnesses, healthier behaviors, and a spouse/partner were more likely to maintain a high-increased well-being trajectory during the transition to retirement. Our findings further showed the critical importance of volunteering in enhancing subjective well-being after retirement.

**Discussion:**

This study highlights the importance of understanding distinct well-being trajectories and how resources differently influence retirement stages in Taiwan’s unique context. Policymakers should recognize this heterogeneity rather than implementing one-size-fits-all solutions. Given the crucial role of family support in early retirement and the growing importance of volunteering later, policies should strengthen family systems while expanding social participation opportunities.

## Introduction

Exploring “well-being” in aging and retirement research is critically important, especially under the context of a globally rapid-growth aging population. According to the definition of the World Health Organization, health should extend beyond the mere absence of disease, encompassing complete physical, mental, and social well-being (World Health Organization, 2015). Therefore, subjective well-being, often measured by life satisfaction, is a pivotal measure representing an individual’s overall evaluation of life and essential in understanding and promoting healthy aging.

Transition into retirement significantly impacts individuals’ subjective well-being. This life course transition fundamentally reshapes multiple aspects of life. Various theoretical frameworks have highlighted potential risks associated with this transition. Role theory views retirement as a crisis of losing work roles (Riley and Riley, 1994), while continuity theory suggests that maintaining self-identity may lead to declined well-being (Atchley, 1971). Life course and conservation of resources theories further emphasize how pre-retirement experiences and available resources affect adaptation outcomes (Elder and Johnson, 2018; Hobfoll, 2002). These theoretical perspectives highlight the critical nature of successful retirement adaptation, as difficulties in this transition process can lead to adverse health outcomes (Henning et al., 2016; Wang et al., 2011). Notably, retirement adaptation exhibits considerable heterogeneity, with individuals showing markedly different patterns of adjustment and well-being changes during this transition.

### Comparisons for retirement adaptation trajectories

Research on retirement adaptation trajectories has revealed considerable variability across countries. Studies in the United States have identified three main trajectories. The predominant stability pattern (69–74%) appears among individuals with bridge employment and proactive retirement planning, reflecting the importance of personal preparation in this system. The small recovery pattern (approximately 4%) occurs among those leaving high-stress occupations, suggesting that for a minority, retirement provides relief from demanding work environments. The U-shaped pattern (22–27%) shows initial vulnerability during the retirement transition, particularly among those with health problems or unexpected early retirement, followed by adaptation and recovery through compensatory strategies (Wang, 2007).

A German study also identified three distinct trajectories (Pinquart and Schindler, 2007). The predominant maintaining pattern (76%) shows little change during the retirement transition, followed by a gradual decline, typically observed among married retirees with good health status. The increasing pattern (15%) demonstrates significant improvement in retirement transition, comprising younger males (average age 59.5) with lower socioeconomic status and high unemployment before retirement, suggesting that Germany’s welfare system effectively supports vulnerable workers through the immediate retirement transition. The declining pattern (9%) exhibits a substantial satisfaction decrease at retirement, followed by subsequent recovery, which is more common among older retirees (average age 63) with higher proportions of women (56.7%) and poorer health. Notably, unlike the US stability pattern, which tends to maintain consistent well-being levels, two of three German trajectories show a tendency toward gradual decline in the long term.

However, Australian research identified four distinct trajectories in the retirement context. The maintaining high-satisfaction group (40%) predominantly comprises older, educated males with good health and partner support. Another two were decreasing patterns: a group starting with high satisfaction but gradually declining (28%) and a group starting with low satisfaction that continued to decrease (18%). The former comprises higher proportions of women with lower education and poorer health, and the latter is characterized by younger individuals forced into retirement without employment or partner relationships. The fourth was the increasing-satisfaction group (14%), which consists of younger, educated males with good health but lower social support (Heybroek et al., 2015).

Although varying retirement systems, Western studies consistently reveal three or more adaptation patterns across countries with different proportions and shapes. The stability trajectory predominates in the above nations (40% in Australia to 76% in Germany), whereas the increasing trajectories represent a smaller but consistent pattern (4–15%). The decreasing trajectory shows the most cross-national variation—appearing as a U-shape in the United States, a simple decline in Germany, and two distinct patterns in Australia.

These variations reflect each country’s institutional system. The US emphasizes individual responsibility and private pensions more, benefiting those with adequate planning and bridge employment. Germany’s welfare system provides stronger initial support that may diminish over time. Australia’s mandatory superannuation system creates more systematic lifetime financial accumulation, possibly explaining its more diverse trajectory patterns than the US market-oriented and German welfare-state models.

### The resource-based dynamic model

Despite previous studies emphasizing different factors influencing trajectory membership, they collectively highlight the importance of resources in shaping adaptation trajectories. The resource-based dynamic model provides an integrative framework particularly suitable for understanding retirement adaptation. Drawing from Hobfoll (2002) conservation of resources theory, this model defines resources as individuals’ overall capacity to fulfill their core values and needs. It emphasizes how resource gains and losses dynamically influence well-being throughout the retirement transition (Hobfoll, 2002; Wang et al., 2011).

The model identifies six types of critical resources that fluctuate during retirement transition: physical resources, financial resources, social resources, emotional resources, cognitive resources, and motivational resources.

Physical and financial resources have consistently been linked to retirees’ well-being. Physical resources, including subjective and objective health status, are crucial in retirement adaptation and higher life satisfaction (Barbosa et al., 2016; Yeung, 2018). Research has identified financial resources as the strongest predictor of successful adjustment during the retirement transition (Yeung, 2018). Social and emotional resources impact retirement adaptation differently. Social resources provide informational and practical assistance through family, friends, and partners. However, previous studies showed inconsistent effects and indicated these might be culturally dependent (Barbosa et al., 2016; Yeung, 2018). Emotional resources, including emotional regulation abilities, positive attitudes, and received emotional support, consistently enhance retirees’ adaptability through improved stability, stress management, and optimism (Blekesaune and Hansen, 2021).

Cognitive resources show inconsistent effects—some studies find retirement slightly decreases memory function (Celia et al., 2021), while others report weak or no relationship with adjustment (Allerhand et al., 2014; Hansson et al., 2018). Motivational resources consistently predict successful adaptation, with self-control and flexible goal adjustment helping retirees navigate challenges effectively (Barbosa et al., 2016; Heyl et al., 2007). Together, these diverse resources offer a comprehensive framework for understanding the multiple factors influencing retirement adaptation, highlighting the specific contributions of each resource type. However, while previous studies have explored some of these factors, they have largely overlooked the dynamic nature of these resources across different retirement stages. Specifically, they fail to identify which resources play crucial roles in maintaining high well-being after retirement, which is most important during the transition period, and how the relative importance of these resources shifts throughout the retirement process.

### Resource adaptation patterns in Taiwan’s context

Given the dynamic nature of retirement resources and the influence on adaptation trajectories, Taiwan presents a fascinating case study due to its distinct institutional, economic, social, and cultural context. In other words, Taiwan’s retirement context differs fundamentally from Western countries across several key dimensions. The institutional disparities between Taiwan and Western retirement systems significantly impact resource accumulation patterns.

Western nations generally possess more established and comprehensive pension systems facilitating consistent retirement resource accumulation throughout working careers. Conversely, Taiwan’s retirement systems developed asymmetrically across occupational sectors and emerged relatively late. Under Taiwan’s historical context, retirement benefits emerged in 1943, exclusively for civil servants, military personnel, and teachers—key ruling party supporters. However, most of Taiwan’s workforce consists of private sector laborers, which remained uncovered until the Labor Standards Act (1984) and National Pension (2008) were implemented.

This staggered implementation has created considerable occupational fragmentation with limited system integration and portability. Consequently, the existing structural configuration places many Taiwanese retirees at a significant disadvantage—particularly those from the private sector or those who have navigated multiple career transitions. This situation results in reduced periods for resource accumulation and leaves them with insufficient retirement funds, severely impacting their financial security in later life. Furthermore, Taiwan’s labor pension systems are characterized by significantly lower contribution rates and limited investment options, severely hindering the potential for retirement asset growth. These institutional differences significantly contribute to resource disparities, impacting the financial security of Taiwanese labor retirees. As a result, they may face greater challenges in adapting compared to their Western counterparts (Shen, 2024).

Taiwan’s economic trajectory from 1960 to 1980 also created distinctive occupational patterns. During rapid industrialization, many Taiwanese workers entered labor-intensive manufacturing and heavy industries such as steel, shipbuilding, and petrochemicals. Workers in these sectors faced chronic exposure to high temperatures, hazardous chemicals, and physically demanding labor conditions, resulting in occupational injuries and accelerated physical deterioration. These cumulative health impacts may affect their physical resources and quality of life during retirement. Financially, Taiwan’s export-oriented development strategy necessitated maintaining competitive wages substantially below those of developed nations (manufacturing wages were approximately 1/4 of Japan’s and 1/22 of America’s during the period; Liang and Wang, 2002). This substantial income differential likely limited workers’ lifetime earnings and retirement savings compared to their counterparts in developed economies, potentially constraining their financial resources in retirement.

In addition, Confucian culture socially shapes distinctive retirement dynamics in Taiwan, contrasting sharply with Western individualism. For example, Confucian filial piety expectations foster unique intergenerational interaction patterns, positioning family as a critical source of emotional and social support for retirees (Yasuda et al., 2011). Approximately 60% of older Taiwanese adults lived with adult children in the early 2000s (compared with less than 20% in most Western societies during the 1990s; Yasuda et al., 2011), yielding family support systems that complement formal institutional assistance with medical, financial, and practical aid. Moreover, Taiwan’s small and medium enterprises dominated economy (comprising over 95% of enterprises) created distinctive social networks where many retirees transition from leadership roles to advisory positions within family businesses, maintaining status and purpose through continued engagement rather than complete withdrawal from professional life (Chung and Au, 2021).

Additionally, the Confucian doctrine of the “Middle Way” (zhongyong) further influences retirement adaptation in Taiwan. This philosophy emphasizes balance, moderation, and harmony, encouraging individuals to accept life’s transitions with equanimity rather than resistance. Such cultural orientation guides people to seek inner equilibrium when facing challenges (Ji et al., 2010). For Taiwanese retirees, this embedded cultural value may promote acceptance of role transitions and psychological adaptation even amidst limited material resources, potentially shaping unique well-being trajectories that differ from Western retirement patterns.

### Study aim

This study aims to investigate the heterogeneity of subjective well-being trajectories among middle-aged and older adults during their retirement transition in Taiwan. Specifically, we have two objectives: first, to identify distinct trajectory patterns of subjective well-being during the retirement transition process; second, to examine how five types of resources (physical, cognitive, financial, social, and emotional) influence these trajectories at different transition stages: pre-retirement, transition to retirement, and post-retirement. Although the resource-based dynamic model includes motivational resources, our dataset did not measure this aspect. By conducting this study in Taiwan, our findings can provide insights into retirement adaptation patterns in an Asian cultural context.

### Retirement trajectories hypothese in Taiwan

In light of Taiwan’s unique socioeconomic and cultural context, we propose three distinct well-being trajectories during the retirement transition. Unlike patterns observed in Western literature, we anticipate that the interaction of Confucian family values, the history of rapid economic development, and changing institutional arrangements will generate trajectory patterns that reflect local adaptation strategies.

For the first trajectory, we expect to see a stable but gradually declining well-being pattern, representing most Taiwanese retirees. This trend may reflect traditional values emphasizing harmony and acceptance of life transitions. Although well-being may remain stable during the initial retirement phase, it could gradually decline in later years as retirees encounter inevitable health and financial challenges.

For the second trajectory, we propose a significant increase in well-being among retirees who receive strong family support and actively engage in social activities. Retirement can be transformative, allowing individuals to transition from work obligations to fulfilling roles within their family and community, especially in family-oriented societies. We believe this improvement will be most pronounced for those who seek emotional support from their families and broaden their social connections through volunteering. By participating in these meaningful activities, retirees can rebuild their identity and sense of purpose after leaving the workforce, ultimately leading to a more fulfilling life.

For the third trajectory, we anticipate a decline in well-being among retirees who face resource limitations across various areas. In Taiwan’s fast-changing socioeconomic environment, some retirees may encounter significant challenges when support systems are lacking, especially if they simultaneously deal with health and financial difficulties. We expect this decline in well-being to occur when retirees do not have enough social and family resources to help mitigate the losses related to retirement.

## Methods

### Data source

This study utilized data from the Taiwan Longitudinal Study on Aging (TLSA), a nationally representative survey launched in 1989 through a collaboration between the Taiwan Health Promotion Bureau and the University of Michigan. The TLSA was conducted every 3–4 years, targeting non-institutionalized individuals aged 50 and above, using a three-stage probability sampling method based on Taiwan’s national household registry. The survey aims to monitor the aging process of Taiwan’s middle-aged and elderly populations by assessing their physiological, psychological, and social well-being. Data were collected through face-to-face interviews using structured questionnaires administered by trained interviewers. Nine waves of the TLSA survey have been completed from 1989 to 2019. New cohorts were added in 1996, 2003, and 2015 to mitigate attrition due to deaths and loss of follow-up.

### Study design

This study utilized six waves of the TLSA dataset spanning from 1996 to 2015, as the measurement of subjective well-being was first introduced in 1996 and underwent substantial modification after 2015. We systematically tracked participants’ retirement transitions by collecting observations at three critical time points: pre-retirement, transition, and post-retirement. We defined the transition point as when a participant first began receiving a pension and then verified this information through retrospective analysis of previous wave data to confirm genuine non-retirement status in the preceding period.

**Figure 1:**
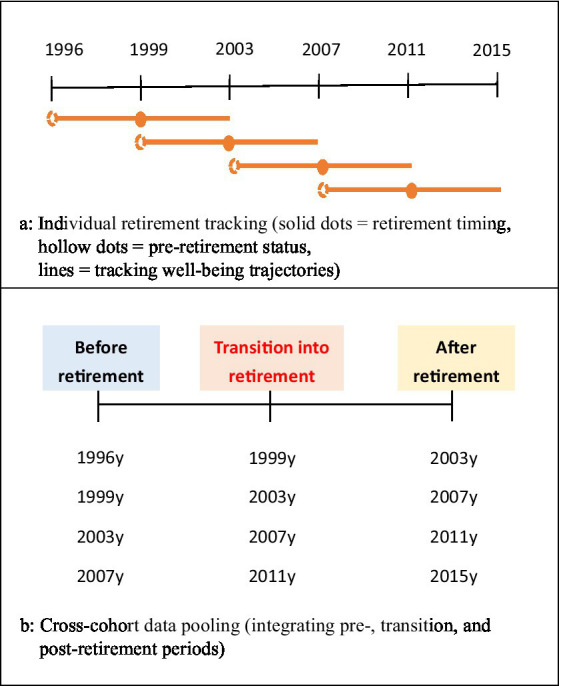
**(A)** Retirement identification and transition tracking method. **(B)** Multi-cohort data integration approach.

Figure 1A shows how to define the retirement status of participants and how to track their well-being trajectories. The solid circles represent the wave in which participants first reported receiving a pension, marking their transition into retirement. The dashed circles indicate the retrospective verification of pre-retirement status. The solid lines represent the tracking of well-being trajectories across pre-retirement, transition, and post-retirement periods.

Figure 1B illustrates how samples from different retirement years were pooled for analysis. The final sample comprises four subgroups of individuals who retired in 1999, 2003, 2007, and 2011. These subgroups were integrated into a unified analytical framework, with each participant contributing an equal number of observations. By including participants from various retirement years, this study simultaneously examines the effects of retirement age and retirement year on the transition and post-retirement phases.

### Participants

**Figure 2:**
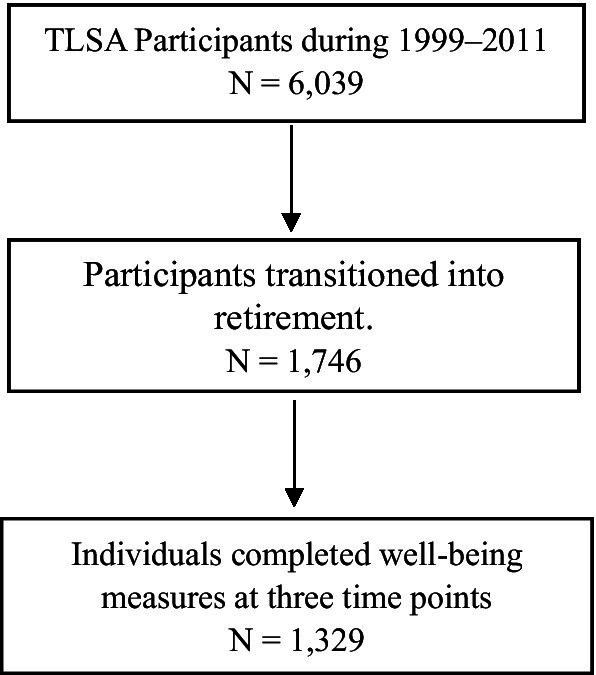
Sample selection process.

This study began with a robust cohort of 6,039 participants from 1999 to 2011, and we meticulously traced their retirement status from one wave before the baseline in 1996 to one wave following 2011 in 2015. This comprehensive approach ensures a thorough understanding of retirement trends over time. As shown in Figure 2, we applied two selection criteria to identify our final analytical sample. The inclusion criteria were as follows: (1) participants had to report receiving a pension during the study period, with confirmed non-pension status in earlier waves (n = 1,746); and (2) they needed to respond to all 10 items of the life satisfaction questionnaire across all three measurement periods: pre-retirement, transition, and post-retirement. Following these criteria, our final analytical sample comprised 1,329 participants aged 50 and above.

### Measurement

#### Subject well-being

Subjective well-being was measured by overall life satisfaction using the 10-item Life Satisfaction Index (Neugarten et al., 1961). This instrument is designed with dichotomous scoring for each item (0 = no, 1 = yes), and a higher score indicates a higher level of life satisfaction. The Cronbach’s alpha in past studies ranges from 0.85 to 0.92.

#### Transition into retirement

Transition into retirement refers to the change in status from non-retired to retired. Retirement status was assessed through self-report in response to the question, “Have you ever received a pension for retirement?” Participants could answer with “Yes” or “No.” A transition was identified when a participant’s status changed from “No” to “Yes” between consecutive waves.

#### Sociodemographic characteristics

Sociodemographic characteristics were assessed as follows. Gender (male, female), education levels (illiterate, literate and primary school, secondary and above), and retirement year (1999, 2003, 2007, 2011) were measured as time-invariant variables. Age was categorized into two groups: 50–64 and ≥65 years. Partner status was determined based on their relationship with their spouse or partner. Those who reported being married or having a cohabiting partner were labeled as “Yes,” while participants who reported being never married, divorced, separated, or widowed were categorized as “No.”

#### Resource status

This study utilized the dynamic resource model, which included six essential resources influencing retirement adjustments. Owing to the absence of suitable questionnaires of motivational resources in the dataset, our analysis incorporated only five of the resources.

##### Physical resources

(a) Self-rated Health:

Participants were asked to rate their current health status, with responses ranging from 5 (very good) to 1 (very poor). We combined “good” and “very good” as well as “poor” and “very poor,” and subsequently categorized them into the following three groups: “Good,” “Fair,” and “Poor.”

(b) Number of Diseases:

Participants were asked to report whether they currently have any diseases from a specified list. This list included hypertension, diabetes, heart disease, stroke, cancer, lung disease, arthritis or rheumatism, liver or gallbladder disease, cataracts, kidney disease (including kidney stones), gout, and hip fractures. We summed each disease reported by each participant, yielding a cumulative disease count for each individual, scoring from 0 to 12.

(c) Number of Unhealthy Behaviors:

Participants were queried regarding their engagement in four specific unhealthy behaviors: smoking, alcohol drinking, betel nut chewing, and not engaging in physical exercise. Responses were coded as 1 for “Yes” and 0 for “No.” We then summed the codes for each unhealthy behavior to produce an unhealthy behavior score for each participant, scoring from 0 to 4.

##### Cognitive resources

Memory recall: Participants were instructed to listen to 10-item objects and then recall and repeat them. The interviewer recorded the number of items each participant accurately recalled. Scores ranged from 1 to 10, with higher scores indicating better memory performance.

##### Financial resources

(a) Full-time Job:

Participants who reported being currently employed in a full-time job were categorized as “Yes.” Those who were temporarily not attending work, assisting with family farming or business, or serving as homemakers were categorized as “No.”

(b) Financial Difficulty:

Participants were asked whether they (and their spouse, if applicable) found it sufficient or challenging to manage their monthly living expenses. Those who reported their financial situation as “quite sufficient with surplus” or “generally sufficient, without feeling a lack” were categorized as having “No difficulty.” Conversely, those who reported “some difficulty” or “considerable difficulty” were categorized as “Yes, experiencing difficulty.”

##### Social resources

(a) Social Activities:

Participants were asked concerning their involvement in various social groups and activities. The types of groups included the following: (1) Community groups, (2) Religious groups, (3) Occupational groups, (4) Political groups, (5) Social service clubs, (6) Hometown or kinship associations, and (7) Senior citizen or study groups. Participants who indicated involvement in any of these groups or activities were classified as “Yes” for social participation; otherwise, they were classified as “No.”

(b) Volunteering:

Participants were asked whether they engaged in any social services or voluntary activities. Based on the responses, they were categorized into the following two groups: “Yes” for those involved in such activities and “No” for those not.

##### Emotional resources

(a) Self-value:

Participants were asked to evaluate themselves as being helpful to their family or relatives with the question, “Generally speaking, do you feel that you are helpful to your family or relatives?” Responses ranged from 1 (not very helpful) to 5 (very helpful). Based on the answers, participants were categorized into two levels: “Not at all or Some,” and “A lot.”

(b) Family Support:

Participants were asked to rate the level of care and concern they received from their family, relatives, and friends. Based on the responses, they were categorized into two levels: “Little or Some” and “A lot.”

### Statistical analysis strategy

We employed the Latent Growth Mixture Model (LGMM) in this study. Using LGMM in trajectory analysis has many advantages. First, unlike traditional growth models, LGMM can identify discrete groups within the same population exhibiting distinct growth trajectories. In addition, LGMM goes beyond latent-class growth models (LGM) by incorporating random effects, allowing for individual variations in growth trajectories over time. Moreover, LGMM can have its unique growth parameters in different latent classes. Lastly, LGMM can effectively manage missing data, utilizing all available observations to compute full information maximum likelihood estimates.

The current study estimated the following multiple models to determine their parameters, shapes, and number of subgroups:

(1) Fixed-intercept models: Assume no growth over time.
(2) Linear growth models: Capture linear changes in fixed components.
(3) Quadratic growth models: Account for nonlinear trends by adding quadratic growth to fixed components.
(4) Linear growth in fixed and mixture components: Address variations within subgroups by including linear growth in both fixed and class-specific components.
(5) Linear and quadratic growth in fixed and mixture components: Incorporate both linear and quadratic growth in both fixed and class-specific components.

Additionally, we considered time random effects in both linear and nonlinear models. Each model, except the fixed-intercept model, was tested with 1–3 classes to capture the growth patterns and potential subgroups thoroughly. We considered several model fit indices to evaluate and select the optimal model.

First, we used entropy values above 0.8 as a primary indicator of high classification accuracy with higher entropy values suggesting greater precision in model classification. Second, we favored the models with lower values on indices such as the Akaike Information Criterion (AIC), Bayesian Information Criterion (BIC), and sample-adjusted Bayesian Information Criterion (SABIC), which indicate effective data representation without overfitting. Third, given the large number of resources considered in this study, we established two criteria for model selection: each class must include at least 20% of the sample to enhance the stability and interpretability of the regression models, and maintain sufficient average latent class posterior probability (ALCPP > 0.8) to ensure classification accuracy. These criteria are interconnected as smaller class sizes often lead to lower ALCPP values, which can compromise the reliability of subsequent analyses (Nylund et al., 2007; Weller et al., 2020).

After determining the best-fitting model, we utilized the Generalized Linear Model (GLM) to identify further predictors influencing the longitudinal trajectories of subjective well-being. All analyses were performed using R statistical software (R Core Team, 2022), utilizing the “lcmm” package for LGMM and the “stats” package for implementing GLM. We also used a grid search approach, conducting 10 iterations starting from 100 random initializations to determine the optimal number of subgroups and achieve the highest likelihood in the multivariate Gaussian mixture models.

## Results

### Characteristics of participants

Table 1 presents the demographic characteristics of the participants. The largest proportion of participants retired in 1999 (42%), followed by 2007 (25%). The sample was predominantly male (73%), with 42% having achieved literacy or a primary school education and 48% having attained secondary education or higher. Approximately 55% began receiving pensions at age 65 or older. The proportion of participants without a spouse or partner increased from 18.2% pre-retirement to 24.2% post-retirement.

**Table 1:** Demographic characteristics of participants at three different time points (*n* = 1,329).

Variables	Pre-retirement	Transition	Post-retirement	*p*-value
	n (%)	n (%)	n (%)	
Retirement year				
1999	–	558 (42.0)	–	
2003	–	251 (18.9)	–	
2007	–	329 (24.8)	–	
2011	–	191 (14.4)	–	
Sex				>0.9
Male	975 (73.4)	–	–	
Female	354 (26.6)	–	–	
Education				>0.9
Illiteracy	132 (9.9)	–	–	
Literary/Primary	563 (42.4)	–	–	
Secondary and above	634 (47.7)	–	–	
Age				**<0.001**
50–64	586 (51.6)	598 (45.0)	371 (27.9)	
65+	550 (48.4)	731 (55.0)	958 (72.1)	
Partner				**<0.001**
No	242 (18.2)	293 (22.0)	321 (24.2)	
Yes	1,087 (81.8)	1,036 (78.0)	1,008 (75.8)	

“–” indicates that demographic characteristics remained constant throughout the retirement transition period. Bold values indicate statistically significant results (*p* < 0.05).

Table 2 illustrates the dynamics of resource status during the observation period. Health metrics revealed a decline in self-rated good health from 50% pre-retirement to 39% post-retirement. The average number of diagnosed diseases increased from 1.23 to 1.94, while cognitive function scores (out of 10) decreased from 5 to 3.4. Unhealthy behaviors slightly decreased from an average of 1.05 to 0.88. Financial difficulties increased from 21% pre-retirement to 24% post-retirement. Both social participation and volunteering experienced minor fluctuations around retirement; however, these changes were not statistically significant. Self-value decreased significantly from 56% pre-retirement to 47% post-retirement, while family support showed a non-significant increase from 86 to 88%. Subjective well-being initially averaged 6.71, increased slightly during the transition, and then decreased to 6.53 post-retirement, though these changes were not statistically significant. Notably, 28% of participants continued full-time work while receiving pension benefits. The proportion reporting financial difficulties was slightly lower before retirement (21%) and increased to 24% after retirement; however, this change was not statistically significant.

**Table 2:** Resource status of participants at three different time points (*n* = 1,329).

Variables	Pre-retirement	Transition	Post-retirement	*p*-value
	n (%)	n (%)	n (%)	
1. Physical Resources				
Self-rated Health				**<0.001**
Poor	239 (18.2)	280 (21.1)	337 (25.5)	
Fair	424 (32.3)	464 (34.9)	474 (35.9)	
Good	650 (49.5)	585 (44.0)	509 (38.6)	
Number of Disease (M ± SD)	1.23 ± 1.34	1.60 ± 1.49	1.94 ± 1.60	**<0.001**
Unhealthy Behaviors (M ± SD)	1.05 ± 0.98	0.92 ± 0.91	0.88 ± 0.87	**<0.001**
2. Cognitive Resources				
Memory recall (M ± SD)	4.95 ± 2.08	4.48 ± 2.10	3.67 ± 2.24	**<0.001**
3. Financial Resources				
Full-time Job				**<0.001**
No	643 (48.4)	959 (72.2)	1,079 (81.2)	
Yes	686 (51.6)	370 (27.8)	250 (18.8)	
Financial Difficulty				0.14
No	1,029 (79.4)	998 (76.8)	954 (76.4)	
Yes	267 (20.6)	302 (23.2)	294 (23.6)	
4. Social Resources				
Social activities				0.3
No	697 (52.4)	661 (49.7)	698 (52.5)	
Yes	632 (47.6)	668 (50.3)	631 (47.5)	
Volunteering				0.11
No	1,187 (89.9)	1,160 (87.3)	1,171 (88.1)	
Yes	134 (10.1)	169 (12.7)	158 (11.9)	
5. Emotional Resources				
Self-value				**<0.001**
None/Some	569 (43.9)	623 (48.0)	663 (53.3)	
A lot	728 (56.1)	675 (52.0)	582 (46.7)	
Family Support				0.5
Little/Some	181 (14.0)	165 (12.7)	155 (12.4)	
A lot	1,116 (86.0)	1,131 (87.3)	1,090 (87.6)	
Well-being (M ± SD)	6.71 ± 2.28	6.78 ± 2.57	6.53 ± 2.86	0.070

Bold values indicate statistically significant results (*p* < 0.05).

### Model fit and selection

Table 3 presents the tests of fit indices and entropy for the LGMM. We examined both fixed-only and fixed-and-random effect models, explicitly focusing on linear and non-linear (quadratic) forms. The table shows the results for two- and three-class groupings, including multiple fitting indicators. We found that all models in the fixed-and-random effects exhibited entropy values lower than 0.8, indicating relatively lower accuracy in predicting group clustering. Thus, those models were abandoned.

**Table 3:** Tests of fit indices and entropy of the growth mixture models.

Models	npm	AIC	BIC	SABIC	Entropy	Group 1 (%)	Group 2 (%)	Group 3 (%)
No growth	3	18,356	18,371	18,362	1.00	100		
Fixed effect models								
Linear	3	18,879	18,895	18,885	1.00	100		
Two class	**6**	**18,060**	**18,091**	**18,072**	**0.81**	**25.4**	**74.6**	
Three class	9	17,969	18,015	17,987	0.79	18.4	71.3	10.2
Quadratic	4	18,878	18,899	18,886	1.00	100		
Two class	**8**	**18,057**	**18,099**	**18,074**	**0.81**	**25.5**	**74.5**	
Three class	**12**	**17,925**	**17,987**	**17,949**	**0.81**	**70.4**	**18.8**	**10.8**
Fixed-and-Random effect models								
Linear	6	18,280	18,312	18,293	1.00	100		
Two class	10	17,882	17,934	17,902	0.67	63.2	36.8	
Three class	14	17,864	17,937	17,892	0.69	63.4	22.3	14.3
Quadratic	7	18,276	18,313	18,291	1.00	100		
Two class	11	17,877	17,934	17,899	0.67	63.2	36.8	
Three class	15	17,859	17,936	17,889	0.69	63.4	22.3	14.3

npm, number of parameters. Bold font indicates candidate models due to higher entropy (0.81). See main text for details.

Three models with fixed effects (two-class linear, two-class quadratic, and three-class quadratic) showed high entropy (0.81). In evaluating these models, we applied three selection criteria. First, we examined group sizes and found that in the three-class quadratic model, Groups 2 and 3 each comprised less than 20% of the sample, making them potentially unstable. Second, we compared fit indices (AIC, BIC, and SABIC) and found that the two-class linear and two-class quadratic models had similar values. Third, considering parsimony, we selected the two-class linear growth mixture model as our final model due to its straightforward structure and interpretability.

### Trajectories of subjective well-being

**Figure 3:**
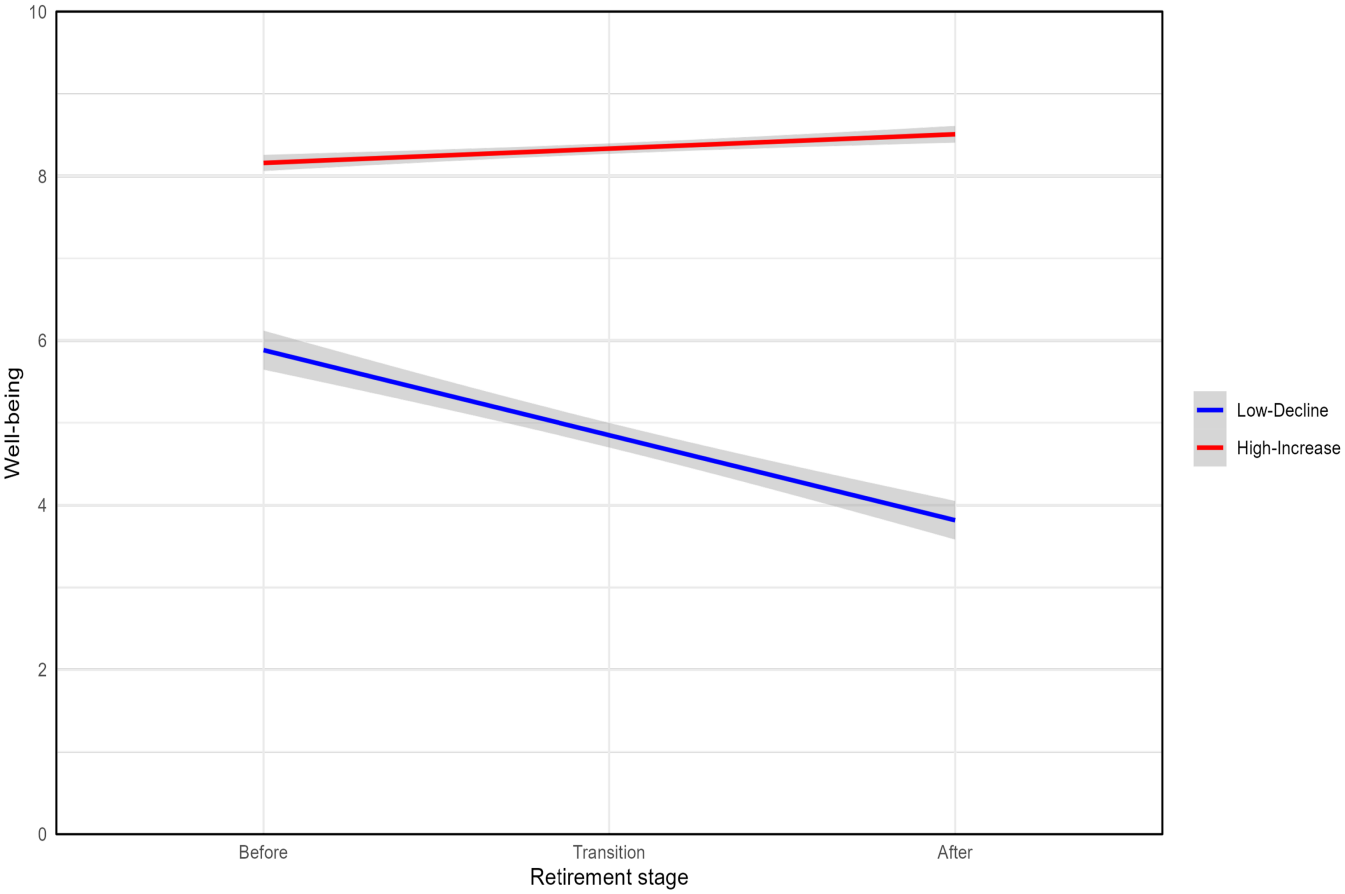
Trajectories of subjective well-being.

Table 4 provides the estimates for the two-class linear growth mixture model. Group 1, which we named the “Low-Decline” group, comprises 25.4% of the sample. This group starts with a relatively low well-being score before retirement and then continuously decreases over time (intercept = 6.02, beta = −0.99, *p* < 0.0001). Group 2, the “High-Increase” group, encompasses 74.6% of the participants. This group begins with a high initial level of well-being and persistently experiences a slight increase as time progresses (intercept = 8.81, beta = 0.22, *p* < 0.0001). Both groups have a high average posterior probability of class membership (≥0.9). Figure 3 shows the linear predictions of the two groups’ trajectories on subjective well-being at three different time points.

**Table 4:** Estimates for linear two-class fixed growth mixture models.

	Low-decline (group 1)	High-increase (group 2)
N (%)	338 (25.4)	991 (74.6)
Average posterior probability of class membership	0.90	0.96
Fixed effects		
Latent intercept (SE)	6.02 (0.12)***	8.38 (0.06)***
Linear time effect (SE)	−0.99 (0.09)***	0.22 (0.05)***

SE, standard error. ***p < 0.001.

### Pre-retirement characteristics of two latent groups

Table 5 shows the demographic and resource distribution of two latent groups. Gender did not differ between these two groups, with the “Low-Decline” and “High-Increase” groups being approximately 74% male and 26% female. The “High-Increase” group is younger, with 55% aged 55–64 years, compared with 42% in the “Low-Decline” group. Additionally, the “High-Increase” group has a higher proportion of individuals with higher education (secondary school and above 53% vs. 34%), having a spouse (85% vs. 72%), full-time job (58% vs. 34%), and no financial difficulties (63% vs. 34%).

**Table 5:** Distribution of pre-retirement characteristics by trajectory groups.

Variables	Low-decline (*N* = 338)	High-increase (*N* = 991)	*p*-value
	n (%)	n (%)	
Sex			0.9
Male	249 (73.7)	726 (73.3)	
Female	89 (26.3)	265 (26.7)	
Age			**<0.001**
50–64	131 (42.0)	455 (55.2)	
65+	181 (58.0)	369 (44.8)	
Education			**<0.001**
Illiteracy	65 (19.2)	67 (6.8)	
Literary/Primary	159 (47.0)	404 (40.8)	
Secondary and higher	114 (33.7)	520 (52.5)	
Partner			**<0.001**
No	94 (27.8)	148 (14.9)	
Yes	244 (72.2)	843 (85.1)	
Self-rated Health			**<0.001**
Poor	125 (37.9)	114 (11.6)	
Fair	96 (29.1)	328 (33.4)	
Good	109 (33.0)	541 (55.0)	
Number of Disease (M ± SD)	1.55 ± 1.57	1.12 ± 1.24	**<0.001**
Unhealthy behaviors (M ± SD)	1.10 ± 0.98	1.03 ± 0.99	0.2
Memory recall (M ± SD)	4.51 ± 2.26	5.09 ± 1.99	**<0.001**
Full-time Job			**<0.001**
No	223 (66.0)	420 (42.4)	
Yes	115 (34.0)	571 (57.6)	
Financial difficulty			**<0.001**
No	200 (62.9)	829 (84.8)	
Yes	118 (37.1)	149 (15.2)	
Social activities			**<0.001**
No	214 (63.3)	483 (48.7)	
Yes	124 (36.7)	508 (51.3)	
Volunteering			**0.002**
No	316 (94.3)	871 (88.3)	
Yes	19 (5.7)	115 (11.7)	
Self-value			**<0.001**
None/Some	58 (18.2)	75 (7.7)	
A lot	261 (81.8)	903 (92.3)	
Family Support			**<0.001**
Little/Some	80 (25.0)	101 (10.3)	
A lot	240 (75.0)	876 (89.7)	
Retirement Year			**<0.001**
1999	157 (46.4)	401 (40.5)	
2003	82 (24.3)	169 (17.1)	
2007	70 (20.7)	259 (26.1)	
2011	29 (8.6)	162 (16.3)	

Bold values indicate statistically significant results (*p* < 0.05).

In terms of health factors, the “High-Increase” group has better-perceived health (55% vs. 33%), fewer diseases (1.12 vs. 1.55), fewer unhealthy behaviors (1.03 vs. 1.10), and higher cognitive function scores (5.09 vs. 4.51). They also have higher participation rates in social activities (51% vs. 37%) and volunteering (12% vs. 6%). Moreover, 92% of the “High-Increase” group feel self-valuable, and 90% received substantial family support, compared with 82 and 75% in the “Low-Decline” group.

### Predictors of subjective well-being trajectories

Compared with the “Low-Decline” group, the “High-Increase” group consistently showed some significant predictors across the three stages. These predictors included higher education levels (OR = 1.73–2.11, 1.65–2.12, 1.77–2.17, respectively), better self-rated health (OR = 2.82–3.64, 1.89–3.55, 2.76–3.17, respectively), fewer financial difficulties (OR = 0.45, 0.33, 0.20, respectively), more social activity participation (OR = 1.43, 1.88, 1.50, respectively), and more substantial family support (OR = 1.65, 2.20, 2.26, respectively).

However, some factors were only presented in specific stages of the retirement transition. For example, in the pre-retirement stage, compared with the “Low-Decline” group, significant predictors of the “High-Increase” group included having a full-time job (OR = 1.50) and possessing high self-perceived values (OR = 1.38). In the transition stage, some factors became significant, including having a partner (OR = 1.56), fewer diseases (OR = 0.85), and fewer unhealthy behaviors (OR = 0.81). In the post-retirement stage, volunteering (OR = 2.24) emerged as a significant predictor of the “High-Increase” group. Notably, gender, age, and memory recall were not associated with subjective well-being in any stage of retirement transition. Additionally, based on our results, retirees in 2011 were more likely to report increased well-being (OR = 2.04), indicating that the most recent retirees enjoyed higher levels of subjective well-being (Table 6).

**Table 6:** Logistic regression models for predicting the membership.

Variables (ref = “Low-Decline” group)	Pre-retirement		Transition		Post-retirement	
	OR	95%CI	OR	95%CI	OR	95%CI
Sex (ref = Male)						
Female	1.07	0.70, 1.64	1.12	0.75, 1.70	1.10	0.72, 1.69
Age (ref = 50–64)						
65+	0.89	0.61, 1.30	0.95	0.66, 1.37	0.93	0.61, 1.40
Education (ref = Illiteracy)						
Literary/Primary	**1.73**	**1.07, 2.80**	**1.65**	**1.00, 2.70**	**1.77**	**1.03, 3.02**
Secondary and higher	**2.11**	**1.26, 3.54**	**2.12**	**1.24, 3.60**	**2.17**	**1.21, 3.89**
Partner (ref = No)						
Yes	1.34	0.91, 1.96	1.56	1.08, 2.26	1.90	1.32, 2.75
Self-rated Health (ref = Poor)						
Fair	**2.82**	**1.86, 4.30**	**1.89**	**1.30, 2.77**	**2.76**	**1.86, 4.13**
Good	**3.64**	**2.36, 5.65**	**3.55**	**2.31, 5.49**	**3.17**	**2.04, 4.97**
Number of Disease	1.00	0.89, 1.13	0.85	0.76, 0.95	0.88	0.79, 0.98
Unhealthy behaviors	0.90	0.76, 1.07	0.81	0.68, 0.96	0.91	0.75, 1.11
Memory Recall	0.97	0.90, 1.05	0.98	0.91, 1.06	1.04	0.96, 1.14
Full-time Job (ref = No)						
Yes	1.50	1.05, 2.16	1.04	0.71, 1.53	0.70	0.44, 1.10
Financial Difficulty (ref = No)						
Yes	**0.45**	**0.32, 0.65**	**0.33**	**0.24, 0.47**	**0.20**	**0.14, 0.28**
Social Activities (ref = None)						
Yes	**1.43**	**1.03, 1.98**	**1.88**	**1.36, 2.63**	**1.50**	**1.06, 2.11**
Volunteering (ref = No)						
Yes	1.25	0.69, 2.38	1.58	0.89, 2.95	2.24	1.15, 4.72
Self-value (ref = None/Some)						
A lot	1.38	1.00, 1.91	1.49	1.08, 2.06	1.36	0.97, 1.92
Family Support (ref = Little/Some)						
A lot	**1.65**	**1.09, 2.49**	**2.20**	**1.47, 3.29**	**2.26**	**1.47, 3.45**
Retirement Wave (ref = 1999)						
2003			0.92	0.61, 1.41		
2007			1.28	0.84, 1.97		
2011			2.04	1.20, 3.55		
	AIC = 1,076		AIC = 1,115		AIC = 983	

Bold values indicate statistically significant results (*p* < 0.05).

## Discussion

This study used a latent growth mixture model to differentiate the relationships between the trajectories of subjective well-being and the experiences with changing retirement resources. We explored life experiences at three stages, pre-retirement, transition into retirement, and post-retirement, to understand how various factors contribute to successful retirement transitions.

### Heterogeneity in subjective well-being trajectories

Our study identified only two distinct linear trajectories of subjective well-being among Taiwanese retirees: a “High-Increase” group (75%) and a “Low-Decline” group (25%). This finding partially aligns with our hypothesized patterns, though with fewer distinct trajectories than initially anticipated. While Western studies typically identify three or more trajectory groups (Heybroek et al., 2015; Pinquart and Schindler, 2009; Wang, 2007), our results suggest a more polarized pattern of retirement adaptation in Taiwan’s sociocultural context.

The predominant “High-Increase” group combines elements of our hypothesized “Stable but Gradually Declining” and “Increasing Well-being” patterns. Contrary to our expectation that most retirees would experience stability followed by a gradual decline, we found that most Taiwanese retirees demonstrated modest improvement in subjective well-being during and after retirement. This suggests that the influence of family-centered support structures and traditional “Middle Way” (zhongyong) values in Taiwan may provide stronger protective effects than anticipated, enabling most retirees to experience retirement as a positive life transition rather than a challenging one.

The “Low-Decline” group (25%) aligns with our hypothesized “Decreasing Well-being” pattern. These retirees experienced deteriorating subjective well-being, confirming our hypothesis that a subset of Taiwanese retirees face significant challenges when they lack sufficient resources across multiple domains. This finding highlights the vulnerability of certain retirees in Taiwan’s rapidly transforming socioeconomic landscape, particularly when traditional support systems are unavailable.

Our supplementary analyses explored a three-class model with “Low-Increase” (10.8%), “Low-Decline” (18.8%), and “High-Stability” (70.4%) groups. However, methodological considerations, including classification precision and sample size stability, led us to adopt the more robust two-class solution. The consistency between our two-class findings and the hypothesized patterns suggests that while the specific number of trajectories differs, the data supports the underlying adaptation mechanisms we proposed (refer to the Supplementary file).

### Resources influencing well-being trajectories

Our findings support the hypothesized framework regarding how different resources influence well-being trajectories across the three transition stages. Particularly noteworthy is how the relative importance of various resources shifted during retirement, revealing distinct adaptation patterns characteristic of Taiwan’s unique context.

Family emotional resources emerged as critical during the retirement transition, strengthening their influence compared with pre-retirement. Having a partner significantly increases the likelihood of positive well-being. Specifically, it enhances this likelihood by 16% from pre-retirement to the transition phase and by an additional 22% from the transition phase to post-retirement. Similarly, family support boosts this likelihood by 33% during the transition and continues to have a strong influence afterward. These findings support our central hypothesis that in Taiwan’s family-centered cultural context, emotional support from family members serves as a crucial buffer against retirement-related challenges, potentially compensating for limitations in other resources.

We have identified a significant transformation in the sources of family support and social engagement during retirement. In the early stages, the perceived self-worth derived from family was crucial in enhancing well-being; however, this influence diminished in later stages. In contrast, the impact of volunteering surged, accounting for approximately 42% of the positive changes observed. This compelling shift underscores our hypothesis about the transition from reliance on family support to a deeper engagement with the community. It highlights that successful adaptation to retirement in Taiwan is not only about receiving familial support but also about actively participating in social activities. This evolution marks a powerful reconstruction of identity and sources of value after leaving the workforce, underscoring the importance of community involvement in shaping a fulfilling retirement (Haslam et al., 2018; Van Solinge and Henkens, 2008).

The changing role of employment status provided further support for our theoretical framework. While full-time employment strongly predicted higher well-being before retirement, this effect disappeared during and after the transition (Heybroek et al., 2015; Pinquart and Schindler, 2007). This finding challenges traditional continuity theory, which suggests that maintaining pre-retirement activities and roles is essential for successful adaptation. Instead, our results indicate that well-being trajectories among Taiwanese retirees are primarily influenced by emotional and social resource allocation rather than occupational continuity.

Physical and cognitive resources demonstrated varied patterns in their influence on well-being trajectories. Health showed dynamic changes consistent with our resource allocation framework, with retirees having fewer chronic illnesses experiencing approximately 15% greater well-being advantages during the transition period compared to pre-retirement. This highlights the critical role of physical health in successful adaptation. Education level, reflecting a life course perspective of cumulative advantage, consistently predicted well-being trajectories across all three-time points, with its influence remaining stable throughout retirement. This stability underscores education’s enduring protective effect. Interestingly, cognitive resources failed to independently predict well-being trajectories after controlling for education, suggesting that education level may be capturing the influence of cognitive abilities (Lövdén et al., 2020).

The historical context of retirement timing revealed an important temporal trend. The later the retirement year, the greater the likelihood of belonging to the “High-Increase” group, with a clear dose–response effect across retirement cohorts from 1999 to 2011. This finding reflects the evolving retirement landscape in Taiwan, with more recent retirees benefiting from improved institutional arrangements and support systems (Drewelies et al., 2019; Gerstorf et al., 2020).

Overall, our findings regarding resource changes across three time points strongly support our hypothesis that in Taiwan’s unique sociocultural context, family emotional resources and social participation play decisive roles in successful retirement adaptation. Despite the economic challenges often associated with retirement, these social and emotional factors remain crucial determinants of well-being trajectories in Taiwan’s family-centered society.

### Study strengths

This study showed several important strengths that combine theoretical foundations with methodological rigor. First, grounded in the resource-based dynamic model of retirement adjustment, we comprehensively explored five types of resources—physical, financial, cognitive, emotional, and social—across three critical stages: pre-retirement, transition to retirement, and post-retirement. This theoretically driven, stage-sensitive approach allowed us to systematically examine how different resource types influence well-being trajectories and how their relative importance shifts across retirement phases. It offers a dynamic understanding of adaptation processes within Taiwan’s unique cultural context.

Second, we utilized longitudinal data from a representative sample of Taiwanese adults, which is particularly valuable for testing our theoretical propositions about retirement trajectories. Compared with other convenience or regional samples, our study used a nationally representative sample, providing more generalizable findings and minimizing sample selection bias. Third, employing latent growth mixture modeling (LGMM) allowed us to empirically identify distinct patterns of subjective well-being changes over time. This advanced modeling technique captured different subgroup trends through unique intercepts and slopes, providing a nuanced understanding of the diverse experiences of retirees that simple aggregate analyses might miss. Lastly, our measurement of subjective well-being using a well-constructed scale rather than a single item improved the reliability and validity of our dependent variable, allowing for more precise trajectory identification.

These strengths powerfully emphasize the theoretical richness and empirical rigor of our study, creating a compelling foundation for understanding retirement adaptation within diverse cultural contexts.

### Study limitations

Although this study provides valuable insights into the trajectories of subjective well-being around retirement, it still faces several limitations due to the constraints of the data source. The Taiwan Longitudinal Study on Aging (TLSA) was not specifically designed to examine retirement transitions and well-being, thus lacking several important predictors. For instance, the dataset did not include key psychological variables, reasons for retirement (particularly whether retirement was due to poor health), pre-retirement working conditions, or the degree of control over retirement decisions. These missing factors could significantly influence well-being trajectories, as research has shown that work mastery affects pre-retirement outcomes and adaptation (Muratore and Earl, 2015), involuntary retirement impacts adjustment and satisfaction (Van Solinge and Henkens, 2008), and job types can moderate effects on subjective well-being (Henning et al., 2022). Thus, future research is suggested to consider the mechanism between these factors and resource changes to explore how they influence well-being trajectories.

The second limitation of this study arises from the intervals between data collection waves, as the survey was conducted every 4 years from 1996 to 2015. These gaps challenge our ability to precisely determine the exact timing of retirement, influencing the assessment of adjustment statuses. For instance, although all participants reported receiving a pension at the second survey point, retirement could have occurred immediately after the first survey or shortly before the second. This timing variation can result in different experiences and adjustments among participants, which are not accurately captured due to the timing of our surveys. This issue highlights the need for more frequent data collection or implementing a time-sensitive tracking method in future studies.

Third, our measurement of resources, while following common approaches in retirement research, captures only partial aspects of these theoretical constructs. For instance, our cognitive resource measure using memory tests, although widely used in previous studies (e.g., Celia et al., 2021; Hansson et al., 2018), represents just one dimension of cognitive functioning. Additionally, while having a spouse emerged as a significant factor in our study and could be considered a form of social resource, it cannot fully capture the complexity of social capital. Future studies should consider including broader measures such as social network size, interaction frequency, and relationship quality, as well as various aspects of cognitive functioning beyond memory tests.

Finally, the two identified retirement trajectories may not universally apply to other countries due to differences in cultural contexts and retirement policies. Nevertheless, our findings support the general principle that retirement adjustment is not a singular pathway or static state. Instead, it is a dynamic and ongoing process of transformation and adjustment, where different resources play varying roles across different stages. This insight highlights the importance of national and cultural heterogeneity in retirement research, especially in Asian countries where retirement research is relatively scarce.

### Practical implications and policy recommendations

Based on our findings, we propose several important policy implications. Despite Taiwan’s decreasing family size and the rise of single- or double-person households driven by rapid aging and low birth rates, our findings underscore the irreplaceable value of family emotional resources during the transition into retirement. Therefore, it is imperative that policies should focus on enhancing family support systems instead of relying on the assumption that institutional care can fully substitute for the vital roles traditionally played by families. Initiatives that promote intergenerational communication, empower family caregivers, and strengthen family bonds are essential to preserving these crucial emotional supports during the pivotal early retirement phase.

Second, our findings highlight the critical role of social engagement throughout retirement, with volunteering becoming increasingly significant in later stages. Policy initiatives should expand accessible opportunities for meaningful community participation and structured volunteering programs that accommodate varying health conditions, allowing retirees to maintain social connections and continue making valuable contributions to society.

Third, our findings challenge current international trends promoting extended workforce participation among older adults. While many countries address labor shortages by encouraging post-retirement employment, our results show that continuing full-time work had no significant positive effect on well-being. This suggests that the nature of work itself may not always be enjoyable; instead, policies should focus on creating voluntary and meaningful civic engagement activities that maintain productive value.

Fourth, early resource accumulation during middle age remains crucial, as our results demonstrate that initial resource levels significantly determine subsequent well-being trajectories. Therefore, policies should prioritize supporting diverse resource development during working years, including promoting higher education opportunities, stable employment, financial security, and programs that enhance self-worth and personal development.

By implementing these recommendations, societies can better address the challenges of demographic transitions while leveraging the essential role of emotional and social resources that our research identifies as key factors in maintaining high life satisfaction throughout retirement.

### Lacking of contribution part

#### Contribution

This research makes several important contributions to retirement literature. First, this study advances the resource-based dynamic model by empirically identifying key predictive resources across three critical stages: pre-retirement, retirement transition, and post-retirement, determining which resources are most crucial when examined simultaneously. Second, our study compellingly highlights the significant differences in retirement adaptation patterns across cultural contexts. Notably, Taiwan exhibits two distinct trajectories, starkly contrasting the three or more commonly found in Western societies. This finding reveals how family structure and cultural values significantly shape retirement experiences. In Taiwan’s unique context, family emotional resources serve as critical buffers during the transition period, while social participation through volunteering becomes increasingly essential later. This pattern reflects Taiwan’s family-centered values and the importance of maintaining social contribution in later life. Fourth, our results show how Taiwan’s institutional context influences adaptation, with the dose-response effect across retirement cohorts illustrating how evolving retirement systems have improved outcomes for recent retirees.

## Conclusion

This study reveals distinctive retirement adaptation patterns in Taiwan characterized by two well-being trajectories—"High-Increase” (75%) and “Low-Decline” (25%). While economic resources maintain consistent importance across all transition stages, family emotional resources become more crucial during transition, followed by social resources growing increasingly influential in later stages. This temporal transformation reflects Taiwan’s unique cultural context, where successful adaptation involves leveraging family networks and reconstructing identity through community engagement. Policymakers should focus on strengthening family support systems during early retirement while expanding opportunities for meaningful social participation later. Our research contributes to a culturally-informed understanding of retirement adaptation relevant to East Asian societies.

## Data Availability

The datasets presented in this article are not readily available due to privacy and confidentiality constraints imposed by the data-collecting organization. Requests to access the datasets should be directed to jenny13929@gmail.com.
