# Supplementary material for: Well-being trajectories and dynamic resource shifts in the transitions of retirement: a longitudinal study of Taiwanese older adults
Source: Front Psychol. 2025 Jun 27;16:1449442. doi: 10.3389/fpsyg.2025.1449442 (PMC12247850; doi:10.3389/fpsyg.2025.1449442)
Supplement: Supplementary file 1 [file Table_1.DOCX]

**Sensitive analysis**

To ensure the statistical robustness of our trajectory findings, we conducted supplementary analyses using a three-class model, which identified "Low-Increase" (10.8%), "Low-Decline" (18.8%), and "High-Stability" (70.4%) groups. Although the three-class quadratic model showed acceptable model fit indices, this solution presented two significant statistical limitations: the smaller groups fell below our predetermined 20% threshold necessary for stable subsequent analyses, and the smallest group showed insufficient classification precision with an average latent class posterior probability below 0.8.

Cross-validation between two- and three-class models further validated our decision: while the main groups demonstrated high classification consistency (99.9% and 95.6%), the smallest group showed substantial instability, with 68.5% being classified into the "Low-Decline" group and 31.5% into the "High-Increase" group in two-class solution. These methodologically rigorous findings provide new insights into retirement adaptation patterns in the Taiwanese context, highlighting the importance of considering both statistical robustness and cultural contexts.

Table S1 Growth mixture models (GMM) estimates: non-linear three group

|  | Low-Decline | Low-Increase | High-Stability |
| --- | --- | --- | --- |
| N (%) | 143 (10.8) | 250 (18.8) | 936 (70.4) |
| Average posterior probability of class membership | 0.76 | 0.87 | 0.95 |
| Fixed effects |  |  |  |
| Latent intercept (SE) | 5.337 (0.25)*** | 6.41 (0.16)*** | 8.48 (0.07)*** |
| Linear time effect (SE) | -2.42 (0.67)*** | 0.50 (0.37) | 0.61 (0.17)*** |
| Quadratic time effect (SE) | 1.68 (0.33)*** | -1.14 (0.19)*** | -0.21 (0.08)*** |
| SE=standard error  ***p<0.001 | | | |

|  | | 3-class non-linear model | | |
| --- | --- | --- | --- | --- |
|  |  | Low-Decline | Low-Increase | High-Stability |
| 2-class linear model | Low-Decline | 98 (68.5%) | 239 | 1 |
|  | High-Increase | 45 (31.5%) | 11 | 935 |
